# Electrochemical (Bio)Sensors as Promising Analytical Tools in the Analysis of Soils, Plants and Environmental Monitoring

**DOI:** 10.3390/bios16050241

**Published:** 2026-04-24

**Authors:** Stella Girousi

**Affiliations:** Laboratory of Analytical Chemistry, School of Chemistry, Aristotle University of Thessaloniki, 54124 Thessaloniki, Greece; girousi@chem.auth.gr

The present Special Issue, entitled “Electrochemical (Bio)Sensors as Promising Analytical Tools in the Analysis of Soils, Plants and Environmental Monitoring”, aims to provide an up-to-date overview of recent advances in electroanalytical techniques and electrochemical (bio)sensors, with particular emphasis on their applications in environmental systems, agriculture, and biological matrices.

Electrochemical sensors and biosensors have emerged as powerful analytical tools due to their inherent advantages, including high sensitivity, selectivity, rapid response, cost-effectiveness, and potential for miniaturization and on-site analysis. These characteristics make them especially suitable for monitoring complex environmental samples such as soils, plants, water, and food systems. Their adaptability further enables integration with modern technologies, paving the way for smart, portable, and real-time monitoring platforms.

This Special Issue brings together contributions from distinguished scientists worldwide, highlighting both fundamental developments and practical applications. A total of eleven papers have been published, including seven review articles and four original research articles, reflecting the dynamic and interdisciplinary nature of the field. Since its publication, the issue has attracted significant attention, with more than 23,000 views and numerous citations, demonstrating the growing interest in electrochemical sensing technologies.

The published works cover a broad spectrum of applications in soil, plants and environmental monitoring. These include the development of electrochemical sensors for assessing soil health through biomarker detection, innovative platforms for monitoring biofilm formation and pathogenic activity, and rapid detection systems for foodborne toxins. Notably, advances in nanotechnology-based sensors for plant biomolecule detection highlight their potential in enabling smart and precision agriculture.

Further contributions explore cutting-edge materials such as two-dimensional carbon nanostructures and melanin-inspired systems for pesticide detection and environmental monitoring. The integration of electrochemical devices with smartphones represents another important trend, offering user-friendly and portable solutions for real-time contaminant analysis in agricultural and food systems. Additionally, emerging tools for detecting hazardous compounds, such as polycyclic aromatic hydrocarbons (PAHs), underline the relevance of these technologies in occupational and environmental health.

Collectively, the articles in this Special Issue demonstrate the versatility and transformative potential of electrochemical (bio)sensors across diverse domains, from environmental monitoring to food safety and agricultural sustainability. The convergence of nanotechnology, materials science, and electroanalysis is expected to further accelerate innovation, leading to more robust, sensitive, and field-deployable sensing platforms.

It has been a great honor to serve as Guest Editor for this Special Issue. I would like to express my sincere gratitude to all contributing authors for their high-quality work and to the reviewers for their valuable insights. Their efforts have made this collection a meaningful contribution to the advancement of electrochemical sensing technologies.

